# Perceived barriers, benefits, facilitators, and attitudes of health professionals towards type 2 diabetes management in Oujda, Morocco: a qualitative focus group study

**DOI:** 10.1186/s12939-023-01826-5

**Published:** 2023-02-07

**Authors:** Usman Sani Dankoly, Dirk Vissers, Souad Ben El Mostafa, Abderrahim Ziyyat, Bart Van Rompaey, Paul Van Royen, Abdellatif Maamri

**Affiliations:** 1 Faculty of Medicine and Health Sciences, University of Antwerp, Antwerp, Belgium; 2 Department of Rehabilitation Sciences and Physiotherapy, University of Antwerp, Campus Drie Eiken, Universiteitsplein 1, 2610 Wilrijk, Belgium; 3 Faculty of Sciences, University Mohammed Premier, Oujda, Morocco

**Keywords:** Type 2 diabetes, Morocco, Qualitative research, Health professions

## Abstract

**Background:**

In Morocco, the treatment of type 2 diabetes (T2D) is mainly focused on medication and only 2% of patients are coached towards a healthier lifestyle. In Oujda, Eastern Morocco the prevalence of T2D is 10.2%, and the current trend is alarming, especially for women. Therefore, the aim of this study is to explore healthcare professionals (HCP) views on the perceived barriers and benefits of an integrated care approach in primary healthcare centers (PHCCs) to T2D management in Oujda.

**Methods:**

A qualitative descriptive study using focus groups in 8 PHCCs. This resulted in a sample of 5 doctors and 25 nurses caring for diabetes patients. The transcripts of all conversations were coded to allow for thematic analysis.

**Results:**

The participants mentioned different *barriers to an integrated approach to DM management:*: excessive workload; poor reimbursement policy; lack of staff and equipment; interrupted drug supply; poor working environment; limited referral; gap in the knowledge of general practitioners; health beliefs; poverty; advanced age; gender; the use of psychotropic drugs. *An integrated approach could be facilitated by* simplified electronic records and referrals; uninterrupted free care; staff recruitment; continuous professional development; internships. *Benefits*: structured care; promotion of care in PHCCs; empowerment of self-management.

**Conclusion:**

HCP views reflect the urge to strengthen the management of T2D in PHCCs. There is a need for HCP with expertise in physical activity and nutrition to solve the current gap in the multidisciplinary integrated care approach. The specific local context in this Eastern Moroccan region, with limited resources and remote hard-to-reach rural areas, can contribute to patients’ reluctance to change their lifestyles, and is a challenge to provide care in an efficient and sustainable manner. More research is needed to see how a patient-centered multidisciplinary approach to T2D management can help motivate patients in Morocco to change to a healthier lifestyle.

**Supplementary Information:**

The online version contains supplementary material available at 10.1186/s12939-023-01826-5.

## Introduction

In 2015, worldwide around 415 million people have diabetes and by 2040, this figure is predicted to rise to 642 million people [1]. Type 2 diabetes (T2D) accounts for 90% of the total diabetes burden [2]. In Morocco, as in many Middle Eastern and North African countries, T2D prevalence is increasing at unprecedented rates more than in any part of the world [3]. Physical inactivity and unhealthy dietary habits have contributed to a high prevalence of T2D, up to 10.6% among adult Moroccans between 2017 to 2018 [4]. In Oujda, Eastern Morocco the prevalence of T2D is 10.2% [5] and the current trend is alarming, especially for women [6].

An international diabetes study shows that Moroccan healthcare professionals (HCPs) mainly focus on the monitoring and medical treatment of diabetes. Only 2% of the study population received lifestyle recommendations [7]. According to 2018 World Bank reports, only 6.35 million people (19%) percent of the population are covered by subsidized basic health insurance. Private hospitals are only accessible by money whilst quality of care remains a challenge for public primary healthcare centers (PHCCs) [8].

**Fig 1:**
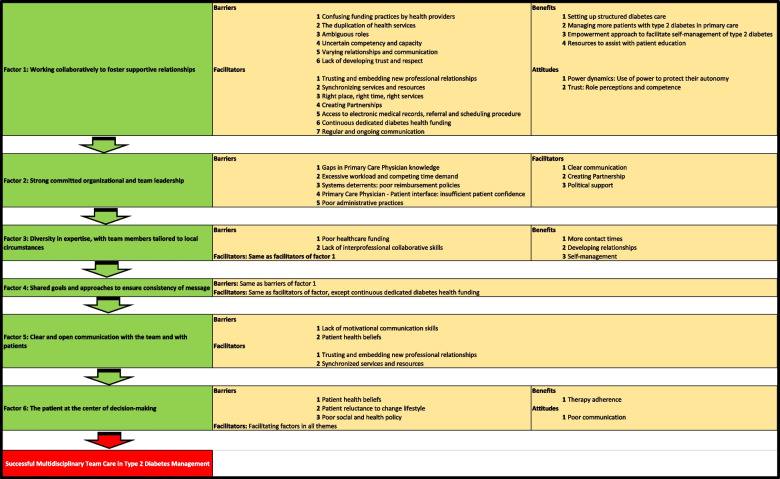
Idea Webbing for textual narrative synthesis. (A higher resolution / colour version of this figure is available in the electronic copy of the article)

Patients with T2D benefit from diverse clinical expertise in integrated multidisciplinary integrated care [9]. Integrated multidisciplinary care means the mechanism for coordinating healthcare services of different professionals to jointly manage patient-specific care. In T2D management, this involves a multidisciplinary team of healthcare professionals that provides behavioral change, diet, insulin therapy, and physical exercise treatments. Our systematic Review [10], finds a huge gap in shared roles among health professionals in T2D therapy and there is a need for allied health professionals, for instance, physiotherapists, dieticians, and psychologists with expertise in diabetes to explore primary healthcare. However, in Oujda, T2D management in public PHCCs is currently limited to general practitioners (GPs) and nurses, and treatment is predominantly focused on medication. Education about healthy food is done by GPs if they have time to do so at all. Exploring the views of HCPs is essential in order to help understand their preferences and suggestions for the improvement of T2D care. In the literature, the views of health professionals about integrated multidisciplinary care in T2D were categorized into six major factors, namely working collaboratively to foster supportive relationships; strong committed organizational and team leadership; diversity in expertise, with team members tailored to local circumstances; shared goals and approaches to ensure consistency of message; clear and open communication with the team and with patients; and the patient at the centre of decision- making. Each of the factors is associated with associated barriers, facilitators, and benefits as illustrated in the Fig. 1 [10]. However, to our knowledge, this is the first qualitative descriptive study that examined the views of the HCPs about this phenomenon in Morocco. The aim of the study is to explore HCPs’ perceptions of barriers, facilitators, attitudes, and benefits towards an integrated care approach of T2D management in Morocco.

## Methodology

### Design

We used a qualitative descriptive research design. Focus groups interviews were used to collect data, as this is an effective way to use group interactions to explore perceptions of HCPs about T2D management [11].

### Settings

The Moroccan health system is composed of a public sector and a private sector (including both for-profit and non-profit providers). The public sector includes 2689 primary health care centres and 144 hospitals at different levels: local, provincial, regional and tertiary. It is a national system spread throughout the kingdom. Primary Health Care is based on the principles of equity and social justice to meet the health needs of all categories of the population through the provision of preventive, curative, promotive and rehabilitative services throughout the life cycle. The duration of the studies of general practitioners is 7 years. The studies are organised in semesters of 5.5 months each and run from the beginning of September to the end of July. The education includes a basic theoretical training and hospital internships. On the other hand, those of the nurses are three and lead to a degree in nursing with the option of multipurpose nurse, midwife, emergency care and so on.

Chronic diseases, including typical diabetes, are managed at the primary health centres. They are monitored by general practitioners and multi-skilled nurses with general training in this area. Medication for diabetics is given free of charge at this level. Type 2 diabetics undergo specialised visits to the regional diabetes reference centre once every 3 months, where they undergo an HBA1c analysis and specialised consultations with the diabetologist/endocrinologist, nephrologist and ophthalmologist. In Morocco, there are no nurses in diabetology but rather multipurpose nurses who undergo basic courses in medical pathology and courses on chronic diseases during their theoretical and practical training. They are assigned to health centres, among other things, for the treatment of diabetics and hypertension.

### Participants

A purposive sample of 30 healthcare professionals (5 doctors and 25 nurses) participated in a focus group interview (*Most nurses* were between 25 and 55 years old while GPs were between 40 and 55 years old. In addition, most participants are women with more than 5 years of work experience. The average age of the participants was 40 years.). Most nurses were between 25 and 55 years old while GPs were between 40 and 55 years old. In addition, most participants are women with more than 5 years of work experience. The average age of the participants was 40 years (Table 1).

**Table 1:** Distribution of HCPs in PHCCs for focus group

Primary Healthcare Centers	Doctors (***n*** = 5)	Nurses (***n*** = 25)
Oued Nachef	0	4
Nssar1	0	3
Sidi yahya	0	4
Al Andalouss	1	1
Mbasso	1	1
Boudir	1	5
Moulay driss	0	4
Lazaret	2	3

Both doctors and nurses had working experience in T2D management for 6 or more months in a primary healthcare center. Besides the clinical task, some of the HCPs have administrative responsibility. Eligibility criteria included having experience of managing patients with T2D and practicing in the health facility where the interview is conducted. The interviews took place in the primary level health centres as this is where chronic diseases including T2DM are managed. Most of the professionals at this level are nurses.

### Procedure

This study was reviewed and approved by the Comité d’Ethique pour la Recherche Biomédicale d’Oujda (CERBO) and participants signed informed consent before participating. An ethics committee approval was received at the beginning of the project under the number CERBO11/19. Anonymity was assured for all participants as no names were used in the transcripts. An informed consent form was completed and signed by each person included in the survey. An interview guide was developed and used to maintain the consistency of methods across groups (Additional file 1). The interview guide was developed and then tested in a health centre with 2 doctors and 4 nurses. Ambiguous questions were rephrased and the quality of the questions was improved. The participants in this test were not included in the sample.

The interviews were done in the French language, but some participants combined this with Arabic dialect in the course of the interview. Arabic dialect means the different linguist spoken in Morocco and some participants combined the dialect with French Language in their conversations.

All local health centers of Oujda were listed. The ones that serve the largest group of patients were asked first to participate, making sure that both rural and urban centers were solicited. T2D management in these settings is currently limited to GPs and nurses. There were no specialist diabetes nurses; instead, the nurses who are responsible for the provision of care for patients with diabetes are called general nurses. Most nurses were between 25 and 55 years old while GPs were between 40 and 55 years old. A separate interview was organized per health center. The interviews took place between November 2019 and February 2020. Each focus group took place either in the doctor’s or medical director’s office of the local health center. Focus group interviews lasted approximately 30 to 120 minutes. More so, the focus group interviews were moderated by the author (AM) who speaks both Arabic and French and is experienced in qualitative research methods.

The focus group interviews were audiotaped and transcribed verbatim into the French language. Initially, all transcripts were written in the French language and before analysis, they were translated into English language using standardized procedures [12].

After interviewing medical staff of 8 PHCCs located in both urban and rural regions in Oujda data saturation was reached. Are between 40 and 55 years old. Each focus group took place either in the doctor’s or medical director’s office.

### Data analysis

The authors (USD, AM, and DV) used the six-phase guide for conducting thematic analysis authored by Braun & Clarke (2006) [13] as a framework for the analysis of the textual data. These include: (I) USD read and re-read transcripts until USD became familiar with the views and language of participants; (II) USD & AM combined inductive and deductive approach to create a thematic framework (III) USD coded the data line-by-line; (IV) USD and AM searched for the themes and emerging sub-themes; (V) AM and DV review the themes; and (VI) USD defined the themes and wrote the results. USD and AM move forward and back within these six-phases in a nonlinearly approach and met via face-to-face meetings to identify the themes, which were discussed until consensus was reached. Transcripts in Microsoft Word format were imported into a qualitative research software package (NVIVO 12 Pro) to facilitate data management and analysis.

## Results

**Fig. 2:**
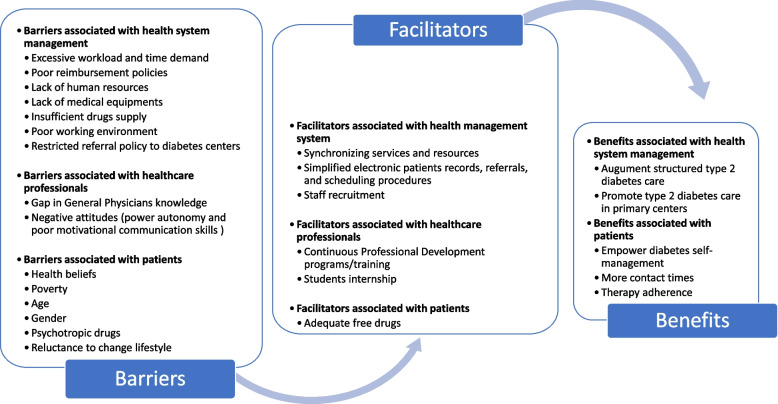
Conceptual framework of type 2 diabetes treatment barriers, facilitators, attitudes and benefits derived from general practitioners and nurses in Oujda

Health professionals identified barriers, facilitators, attitudes, and benefits of T2D treatment in primary health centers, which were grouped into three main categories: healthcare system, HCPs, and patients (Fig. 2).

### Barriers associated with health system management

#### Excessive workload and lack of human resources

The disproportionate clinicians-to-patient ratio was mentioned by the participants as a major problem affecting proper individual medical consultations. They added that only a few doctors and nurses run the diabetes clinic and have to attend to long queues of patients beyond official working hours. Thus, often patients are given longer appointments for medical follow-up.

Too many patients with diabetes are attending consultations, which does not allow adequate centered care for each patient (D).

Diabetic patients and health workers suffer due to too much workload (N).

Lack of enough staff to listen well (N).

We give longer appointments (D).

Nurses have mentioned that one nurse is responsible for multiple tasks, which is time demanding. In addition, participants attribute shortage of staff to lack of recruitment and demotivated staff who are overdue for retirement but still in public service. Besides the lack of general practitioners in PHCCs, the doctors said there are no medical specialists to attend to patients with T2D requiring advanced care, and added that the nurses are not certified, diabetes specialists.

The nurses do not do their job due to lack of time, workload (N).

Staffs are already old, sick, and demotivated (3 years above retirement age) almost 41 years of civil service (N).

Specialists are no longer available in diabetic centers (D).

If a patient is hypo or hyperglycemic, there is no referral to a specialist (N).

#### Restricted referral policy to diabetes centers

The referral system is vague and patients find it difficult to make the transition from PHCCs to specialized healthcare centers. Participants noted that the Centre de Référence Préfectoral Diabétologie et Maladies Chroniques restrict referral of T2D patients to limited figures as opposed to the large number of patients with complications who need specialized care. Nurses mentioned that doctors exercise power autonomy by monopolizing the decision for referring patients to specialized care. More so, nurses perceived that they always had to fill in too much patient information on the computer during consultations. They said it demands too much time and effort, as they have too many patients to attend to.

Centre de Référence Préfectoral Diabétologie et Maladies Chroniques gives us a specific number of patients for referral (N).

Ambiguous referral system and difficult for the patient to identify (D).

It is only the doctors who recommend a referral if a patient is unstable or has complications (N).

Complex database for health record information system (N).

#### Poor reimbursement policies and working environment

At public hospitals, all HCP’s salaries are paid by the Ministry of finance. Doctors perceived their salary as low and not meeting their expectations. Participants described their working environment as having deteriorating working conditions. In addition, they mentioned that the universal medical coverage is inadequate due to lack of medical equipment and often the disrupted supply of free drugs. In addition, necessary medical investigations are left undone due to the poor economic status of the patients.

Lack of motivation in health professionals (D).

Lack of adequate premises and diabetes education materials (N).

The government cannot provide complete medical coverage for all the people. Lack of blood glucose meters. Routine assessment of lipid profile and HbA1c every three months are not done, because it's expensive. Lack of drugs. Patients do not buy drugs, because they are poor. Blood tests are not done and patients abandon their follow-ups (D).

### Barriers associated with healthcare professionals

#### The gap in doctor’s knowledge

General practitioners expressed their incapability to manage elderly patients and complications. Questions about the general knowledge of T2D revealed that doctors perceived T2D as a chronic disease frequently found in young adults and the elderly population, and further highlighted heredity, obesity, and sedentary lifestyle as associated risk factors. They mentioned that treatment is non-insulin-dependent and lifestyle-oriented.

Doctors only control the blood sugar and prescribe medication. When there are complications nothing can be done. They always come with complications (diabetic feet, amputation, or dialysis) (D).

We do not target stable glycemic control in the elderly because it is difficult (D).

Chronic disease, disease of the century. In all age categories above 30 years. Associated with heredity, obesity, or sedentary lifestyle. Clinically, a serious pathology. Requires assessment and multi-discipline monitoring (D).

### Barriers associated with patients

The participants expressed that beliefs about T2D among Moroccan patients are constructed out of cultural values and spiritual beliefs. Several patients believed that any illness including T2D comes from Allah (God) and he decides their fate with or without having T2D. This belief made them less likely to follow lifestyle changes advice. However, they do pay attention to getting free medicines.

Patients do not accept their illness and do not adhere to diet advice, everything. Always the diabetic patients perceive higher HbA1c as caused by stress. They just need the drugs (D).

Participants mentioned that patients’ willingness to adhere to treatment advice is negatively influenced by their poor socioeconomic status. Doctors mentioned the role of gender in terms of adherence to advice in favor of men. Nurses added that patients of advanced age did not engage in physical activity. Nurses said T2D patients were often prescribed antidepressant drugs, it influences their behaviors.

Patients’ adherence to advice depends on socioeconomic status, culture, and education level (D).

Men adhere to advice more than women (D).

Aged have a problem especially with physical activity (N).

Diabetes patients always take psychotropic drugs (N).

### Facilitators and benefits associated with health system management

Participants suggested that an institutionalized referral system to a specialist, a simplified electronic health record, a continuous supply of free drugs, a diagnostic tools referral system, and recruitment of more HCPs into the civil service will augment structured T2D care in primary centers.

Availability of drugs and equipment in adequate quantity and quality. Free routine medical investigations, for example, HbA1c and blood glucose. Recruit young competent staff. Simplify the health record database. Provide nurses just responsible for type 2 diabetes. Reduce appointment times (D).

*You need a nutritionist, dietetics, or nurse at CRD (in case of obesity) …*. *Ensure endocrinology consultation 1 time in 3 months in the primary medical centers* (N)*.* They added that there is a need to improve the working atmosphere. *Favorable working conditions, air conditioning* (N).

### Facilitators and benefits associated to HCPs and patients

Participants recognized the need to engage in lifelong learning. They mentioned attending training would benefit continuously upgrading their skills. On the other hand, the participants perceived the provision of free medical services as an approach that will promote diabetes self-management and therapy adherence. In addition, they stressed the need to use social media to create awareness and to promote diabetes education.

Continuous professional development program for staff that will provide diabetes education (D).

Free medical consultation for these patients. Otherwise, patients will neglect the disease if not provided with their needs, everything (D).

Therapeutic education should be done in the media and social networks for detailed diabetes education (D).

Locally adapted poster and teaching material (N).

## Discussion

This study provided an in-depth understanding of the barriers and facilitators for a integrated approach to T2D management. The barriers were excessive workload, insufficient medical consultation time, and lack of human resources. The views of nurses, doctors, and health service managers differ, so determining the optimal doctor-to-patient ratio is a complex matter [14]. For too many GPs, the workload has become unmanageable, so patients don’t receive adequate care. Moreover, the pressure leads to burn-out and the intention to leave the profession earlier than planned. This will certainly have a major impact on the time patients have to wait for a follow-up medical appointment [15].

Although there is insufficient evidence to either support or resist a policy of increasing consultation lengths, longer consultations may facilitate integrated care and offer sufficient time to explain treatment options to patients [16]. More so, nurses described their work as multitasking demanding a lot of time. Indeed, nurses’ cognitive load is exceptionally heavy and the environment surrounding them is fast-paced and unpredictable. Nurses can manage interruptions and multitask well, but the potential risk for errors increases with the workload, hence strategies to decrease interruptions are needed [17].

Doctors raised concerns about the lack of easily available specialists to attend to patients suffering from complications. Also, they described the referral system as unclear and difficult for patients to make a transition from PHCCs to specialized care. Moreover, only a limited number of patients with T2D are accepted in diabetes referral centers. Literature on the referral practices of GPs for diabetes shows that factors influencing the referral threshold include multiple complications, the presence of comorbidities, uncertainty concerning medical management, and the need for self-management education [18, 19]. The inability to gain timely access to specialized diabetes consultation teams may contribute to GP’s and patients’ frustration and sub-optimal disease management [11].

In this specific study, nurses perceived that it is the sole responsibility of doctors to refer patients to specialized care. However, models of nurse-led T2D clinics exist worldwide. Hence, this needs to be addressed in order to improve the quality of diabetes care. Evidence shows that diabetes nurse specialists have a key role in supporting and coordinating the integrated care management of T2D through nurse-led clinics in primary care. They facilitate the delivery of integrated care within the PHCCs and the cooperation between health care settings and specialists [20, 21]. An integrated approach for T2D management is effective in significantly reducing glycated hemoglobin levels, increasing treatment compliance and it is cost-effective [22]. Including specialist diabetes nurses, dieticians, physiotherapists and psychologists would allow GPs and nurses to focus on their core tasks. GPs and Nurses found it difficult to include physical activity support and healthy lifestyle advice in aT2D care program. But integrating physiotherapists into the interdisciplinary leads to the opportunity to include specialized diabetes physical activity care to elderly patients [23]. In our study, we were not able to explore how GPs and nurses advise patients to engage in physical activity since triangulation between the quotes of nurses and GPs was not done..

The introduction of an electronic health record system (EHR) is an important development to facilitate the processing of large amounts of patient data. However, in our study nurses perceived EHR as an additional workload in T2D care because they had to input numerous data during medical consultations. This finding is quite essential given the current trend of using computer applications in diabetes management, and thus this contributes to the strength of this study. A previous study [24] that examines the emerging practices of the use of EHR in diabetes care reported that there is a mismatch between EHR designs and diabetes care practices. Some health professionals perceived EHR as a source of increase in caregivers’ administrative time, while others described EHR as a tool that modestly decreases the clinical documentation time. Therefore, it is recommended that system designers first make overviews of the necessary information about diabetes patients in order to develop systems that can be used optimally during clinical consultations.

Doctors that participated in the focus group discussions indicated that they were not satisfied with their salaries. Clinical management, according to recommended guidelines, at the level of primary care improves outcomes for patients with diabetes but it is linked to incentives for health professionals. In the United Kingdom, evidence has shown that incentive models have spurred some improvements in process outcomes and achievement of cholesterol, blood pressure, and HbA1C targets [25]. To improve subsidized diabetes care, Morocco has included the prevention and control of diabetes as a priority program in its health action plan 2012–2016. Still, diabetes care is limited. In 2011, the number of diabetic patients supported by the Moroccan Ministry of health was 460,000 representing only 33% of the known diabetics [26]. Patients who have no health care insurance receive high medical bills. Those who cannot afford or are financially unable do not use the health services or do not adhere to the treatment [27]..

Islam is predominantly the religion practiced in Morocco and some Muslims with T2D believe that health and illness are part of Allah’s plan determining their fate and time of death with or without having T2D. Consequently, patients are less likely to clearly understand the disease and the urgency for lifestyle changes. Alsairafi et al., [28] reported that many patients believed that only God can cure diabetes and not doctors or medicines. However, it is well known that severe untreated cases of diabetes can lead to death, and there are different verses of the Holy Quran (the Islamic divine book) and Prophet’s sayings that stress disease prevention and treatment.

Participants in our study perceived that elderly patients do not engage insufficient physical activity. However, there is functional heterogeneity because some older patients are highly functional, some are disabled and frail, and many are between these two extremes [29]. Hence, tailored programs can address this specific population.

### Strengths and limitations

To the best of our knowledge, this study was the first in Morocco to assess perceived barriers, benefits, facilitators, and attitudes of health professionals towards T2D management. Nevertheless, our study has some limitations. A convenience sample was used to select the participants possibly leading to some missed respondents. The data was translated into English affecting the trustworthiness of the original data due to an interpretation of the translator. Also, there was no member checking, and we did not include perceptions of patients. However, the specific findings in this study provide essential information to strengthen T2D care in this region. We also reached data sufficiency after eight focus groups interviews, giving sufficient richness and depth of the data.

## Conclusions and implications

Our findings indicate a need for an adapted patient-centered multidisciplinary team and an integrated care approach. The importance of political will and the possibility to ensure adequate universal health coverage, and well-equipped PHCCs with synchronized secondary care service should be emphasized. In addition, including dieticians, physiotherapists and occupational therapists with expertise in diabetes are crucial for establishing lifestyle-changing interdisciplinary programs. A discussion of our findings with the health policy authorities of the Ministry of Health of Morocco could lead to suggestions to strengthen the organization of T2D care to meet the needs of patients. There is a need for changes in the behavior of professionals towards less authoritarian styles of medical decision-making and more patient-centered care. The characteristics of the patient-care provider relationship that affect reluctance to change lifestyle also deserve further research.

## Supplementary Information

**Additional file 1: **Interview guide.

## Data Availability

The datasets used and/or analysed during the current study are available from the corresponding author on reasonable request.
