# Supplementary material for: Perceived barriers, benefits, facilitators, and attitudes of health professionals towards type 2 diabetes management in Oujda, Morocco: a qualitative focus group study
Source: Int J Equity Health. 2023 Feb 7;22:29. doi: 10.1186/s12939-023-01826-5 (PMC9903508; doi:10.1186/s12939-023-01826-5)
Supplement: Supplementary file 1 — Additional file 1: Interview guide. [file 12939_2023_1826_MOESM1_ESM.docx]

# ADDITIONAL FILE 1: INTERVIEW GUIDE

**FRENCH VERSION**

**Focus group**

Nous avons élaboré un scénario pour la réalisation d'entretiens collectifs de type focus group. Ce canevas de type semi-structuré est préétabli afin d'étudier la variété des opinions des professionnels de santé, d'identifier des idées nouvelles et de mettre en évidence des problématiques qui pourraient être creusées dans le cadre d'une enquête plus approfondie sur le DT2.

La trame des focus groups interviews sera construite afin d'explorer les connaissances des professionnels de santé au niveau des centres de santé. Les expériences personnelles de chacun, les attentes concernant les structures de soins ainsi que les difficultés persistantes à la prise en charge du DT2 seront discutés.

**Site de l’étude**

L'étude aura lieu au niveau de l'ensemble des ESSB de la préfecture d’Oujda Angad, où des patients diabétiques sont pris en charge par des médecins généralistes.

**FOCUS GROUP**

La méthode du Focus Group est une méthode qualitative de recherche sociale qui favorise l'émergence de toutes les opinions. Elle sera réalisée avec des échantillons hétérogènes de professionnels de santé volontaires, hommes et femmes.

- Objectif : Evaluer et analyser les besoins et les attentes des professionnels en matière de PEC du diabète T2
- Les Focus Group seront réalisés en français, dans les locaux des ESSB choisis
- Le nombre de participants par focus group est limité à 6
- La durée moyenne par focus group est de 2 heures.

Voici la trame avec **les 6 grands thèmes** qui seront abordés et les probes préétablis (points importants à aborder) :

1. Que représente le diabète 2 pour vous ? Pas de probes particuliers, chacun doit prendre la parole

2. Quelle est votre expérience personnelle concernant vos patients reconnus diabète type 2 ?

3. Comment jugez vous la prise en charge des diabétiques type 2 actuellement ?

4. Comment jugez-vous le comportement des patients diabétiques vis-à-vis de vos conseils ?

5. Quelles sont les difficultés et obstacles pour l'éducation thérapeutique que vous rencontrez avec vos patients diabétiques de type 2 ? Pas de probe particulier, chacun doit prendre la parole

6. Pour quelles raisons recommandez-vous/ ne recommandez-vous pas une approche pluridisciplinaire à vos patients ? Probes : a. Raisons de recommandations b. Raisons de non recommandations

**ENGLISH VERSION**

**Focus group**

We have developed a scenario for carrying out collective focus group interviews. This semi-structured framework is established a priori in order to study the variety of opinions of health professionals, to identify new ideas and to highlight issues that could be explored in the context of a more in-depth investigation into the type 2 diabetes mellitus (T2DM).

The framework of the focus groups interviews will be constructed in order to explore the knowledge of health professionals at the level of the local health centers. The personal experiences of each person, the expectations concerning the structure of health care provision as well as potential persistent difficulties in the management of T2DM will be discussed.

**Study site**

The study will take place in health care centers in Oujda, Morocco in the Angad prefecture, where diabetic patients are treated by general practitioners.

**FOCUS GROUP**

The Focus Group method is a qualitative method of social research that promotes the emergence of all opinions. It will be carried out with heterogeneous samples of volunteer health professionals, men and women.

• Objective: Evaluate and analyze the needs and expectations of professionals about management of T2DM.

• The Focus Groups interviews will be carried out in French, in the premises of the chosen health care centers

• The number of participants per focus group is limited to 6 persons

• The average duration per focus group is 2 hours.

Here is the framework with the **6 main themes** that will be addressed and the pre-established important points to address:

1. What does T2DM mean to you? No specific sub-themes, everyone must speak

2. What is your personal experience with patients with T2DM?

3. How do you evaluate the current treatment of T2DM you and your patients have access to?

4. How do you evaluate the behavior of diabetic patients towards your advice?

5. What are the difficulties and obstacles for therapeutic education that you encounter with your patients with T2DM? No specific sub-themes, everyone speaks up

6. For what reasons do you / do you not recommend a multidisciplinary approach to your patients? Sub-themes: a. Reasons for recommendations b. Reasons for non-recommendations
